# Comprehensive studies of biological characteristics, phytochemical profiling, and antioxidant activities of two local citrus varieties in China

**DOI:** 10.3389/fnut.2023.1103041

**Published:** 2023-01-25

**Authors:** Lifang Sun, Jianguo Xu, Nasrullah, Luoyun Wang, Zhenpeng Nie, Xiu Huang, Jianhua Sun, Fuzhi Ke

**Affiliations:** ^1^Institute of Citrus Research, Zhejiang Academy of Agricultural Sciences, Taizhou, China; ^2^National Center for Citrus Variety Improvement, Taizhou, China; ^3^Department of Plant Biology and Ecology, College of Life Sciences, Nankai University, Tianjin, China

**Keywords:** Zangju, Tuju, biological characteristics, phytochemicals, antioxidant activity

## Abstract

Citrus is widely grown all over the world, and citrus fruits have long been recognized for their nutritional and medical value for human health. However, some local citrus varieties with potentially important value are still elusive. In the current study, we elucidated the biological characteristics, phylogenetic and phytochemical profiling, antioxidants and antioxidant activities of the two local citrus varieties, namely Zangju and Tuju. The physiological and phylogenetic analysis showed that Zangju fruit has the characteristics of wrinkled skin, higher acidity, and phylogenetically closest to sour mandarin *Citrus sunki*, whereas, Tuju is a kind of red orange with vermilion peel, small fruit and high sugar content, and closely clustered with *Citrus erythrosa*. The phytochemical analysis showed that many nutrition and antioxidant related differentially accumulated metabolites (DAMs) were detected in the peel and pulp of Zangju and Tuju fruits. Furthermore, it was found that the relative abundance of some key flavonoids and phenolic acids, such as tangeritin, sinensetin, diosmetin, nobiletin, and sinapic acid in the peel and pulp of Zangju and Tuju were higher than that in sour range Daidai and satsuma mandarin. Additionally, Zangju pulp and Tuju peel showed the strongest ferric reducing/antioxidant power (FRAP) activity, whereas, Tuju peel and pulp showed the strongest DPPH and ABTS free radical scavenging activities, respectively. Moreover, both the antioxidant activities of peel and pulp were significantly correlated with the contents of total phenols, total flavonoids or ascorbic acid. These results indicate that the two local citrus varieties have certain nutritional and medicinal value and potential beneficial effects on human health. Our findings will also provide an important theoretical basis for further conservation, development and medicinal utilization of Zangju and Tuju.

## Introduction

*Citrus* belong to the family Rutaceae and are considered as one of the largest fruit plant species broadly dispersed in the temperate, tropical, and subtropical areas with potential socio-economic influence (1). Oranges, grapefruits, mandarins, pummelos, lemons, and limes are popular for nutritional value and are the main industrialized citrus crops (2). Citrus fruits, not only their delicious flavors, are also the most abundant fruits containing valuable beneficial phytochemicals and are rich sources of natural antioxidants, which are now widely accepted as being beneficial to human health (2–4). Antioxidants in food have been shown to have a suppressive effect on oxidative stress *in vivo*, and are thought to play a role in the prevention of atherosclerosis and complications from diabetes (5, 6). The antioxidant activity and medicinal value of citrus fruits has been researched and reported in many literatures (3, 7, 8).

Antioxidant activity denotes the capability of a bioactive compound to clear free radicals and inhibit oxidative degradation, such as lipid peroxidation, for preventing the oxidative damage (9, 10). It is the foundation of many biological functions and associated with the prevention of many chronic diseases (3). Therefore, natural antioxidants from fruits play a predominant role in stabilizing the health of human. In recent years, the citrus fruits are attracting more attention due to their potential health-promoting functions, which contain a number of secondary metabolites with antioxidant activity, such as flavonoids, carotenoids, phenolic acids, vitamins, alkaloids, coumarins, limonoids, and essential oils (11). These active secondary metabolites have a wide variety of biological activities of importance to human health, including anti-oxidative, anti-microbial, anti-inflammatory, anti-cancer, anti-proliferative, anti-mutagenicity, anti-carcinogenicity and anti-aging, as well as cardiovascular protective effects, hypoglycemic and insecticidal activities, neuroprotective effects, etc. (3, 11–15). In addition, attributed to the presence of these medicinally active secondary metabolites, citrus fruits have been used as traditional medicinal herbs for relieving stomachache, fever, cardiac diseases, edema, snakebite, bronchitis, and asthma (16) in several Asian countries for a long time, such as China, Japan, and Korea (17).

Many clinical and animal studies have shown that some citrus metabolites can help protect against the effects of reactive oxygen species (ROS), improve digestive function, and prevent cardiovascular diseases, inflammation, diabetes, and neurological diseases (18–20). Different varieties of fruits exhibit a great diversity in secondary metabolite constituents, and antioxidants with different components in citrus fruit extracts contribute unequally to their total antioxidant ability. Much of the total antioxidant activity of fruits is related to their phenolic content, and a close correlation exists between the polyphenol content and antioxidant activities (21). One previous research suggests that many flavonoids are more potential antioxidants than vitamins (22). Accordingly, it has been reported that citrus peels in traditional medicine exhibit important pharmacological and nutraceutical properties, and these bioactivities are significantly related to the amounts of active polyphenols (phenolic), especially phenolic acids and flavonoids (23). The phenolic composition and antioxidant activity of fruit tissues from different mandarin cultivars were reported by two researchers, and it was found that the peel and juice are the main tissues with higher total phenolic content and total antioxidant activity compared to pulp and seeds (24). Logically, DPPH or ABTS values of antioxidant activities showed higher correlation with the phenolic content in different fruit tissues (25). Furthermore, the phenolic compounds and antioxidant activities of different fruit parts and species of citrus were widely evaluated. For example, it was found that the polyphenol contents and antioxidant activities for the different fruit parts of nine grapefruits varieties varied as the following order: flavedo > segment > membrane > juice vesicle > albedo > seed, with a diversity among all the varieties (26). Besides the difference between different tissues of fruit, a remarkable diversity exists in the content of polyphenols and antioxidant activity among citrus varieties. Several previous reports showed that the highest levels of total phenols and total flavonoids were found in mandarin (27). Naringin was the dominant compound in the peel of pummelo (*Citrus grandis*), while mandarin (*Citrus reticulata*) was rich in hesperidin (25, 28, 29). Grapefruits (Cocktail and Rio Red) were more precious than the pummelo in flavonoids (26, 28). The highest phenolic acid content, dominated by protocatechuic acid, was found in kumquat (27). Additionally, Zhang et al. reported that the content and composition of phenolic compounds, including flavonoids and phenolic acid, and antioxidant capacities of 14 native wild mandarin genotypes showed clear differences in the grapefruit or mandarin group (8).

China is one of the important center origins for the genus *Citrus*, and many important citrus genotypes originated from here (30, 31). Over the past few years, many local citrus genotypes in China have been researched and documented. For example, the content and composition of bioactive compounds in 14 Chinese wild mandarin genotypes and 27 local citrus cultivars had been determinated and their antioxidant activities were also evaluated (8, 32). In this study, for the first time we studied the biological characteristics, phylogenetic relationships, phytochemical profiles, and antioxidant activities of the two local citrus varieties in China, Zangju (ZG) and Tuju (TG). The aim of our study is to explore the nutritional and medicinal value and provide an important theoretical basis for the conservation and medicinal utilization of these two local citrus varieties.

## Materials and methods

### Biological characteristics and phylogenetic analysis of Zangju and Tuju

Mature fruits collected from Zangju (Derong county of Sichuan province), Tuju (Chunan county of Zhejiang province), Daidai, satsuma mandarin trees were divided into peel (flavedo and albedo) and pulp (segment epidermis and juice vesicle). Five similar fruits were collected as one repeat, three repeats for each group. For each variety, peel and pulp of more than five fruits collected from different trees were sampled as one repeat and frozen in liquid nitrogen for metabolites extraction and subsequent RNA extractions, three repeats for each group.

Both of the two total genomic DNA were extracted from 100 mg of fresh leaves frozen in liquid nitrogen using a modified CTAB method. The DNA concentration (>50 ng μl^–1^) was measured using a NanoDrop spectrophotometer, and fragmentation was achieved using sonication, and integrity was evaluated using 0.8% agarose gel. Sequencing was performed using an Illumina NovaSeq 6000 platform (Genepioneer Biotechnologies Co. Ltd., Nanjing, China) with PE250 based on Sequencing by Synthesis (SBS) technology. Except for Zangju and Tuju, the chloroplast genomic sequences of the other 20 citrus varieties and 2 close genus were downloaded from NCBI is used for and manually annotated for phylogenetic tree analysis. MAFFTv.5 (33) was utilized to align the cp genomes of the 24 species. Then we constructed a maximum likelihood (ML) tree using MEGA 7, and a bootstrap test was performed with 1,000 repetitions.

### Extraction, identification, and analysis of metabolites

Samples of the peel and pulp of mature fruits of Zangju, Tuju, Daidai, and satsuma mandarin stored at −80°C were used for metabolites extraction. Metabolome analysis was performed by Biomarker Technologies Co., Ltd. (Beijing, China) using non-target liquid chromatography–mass spectrometry (LC-MS; Biomarker Technologies) to identify differences in the metabolite profile among treatments. The LC/MS system for metabolomics analysis is composed of Waters Acquity I-Class PLUS ultra-high performance liquid tandem Waters Xevo G2-XS QT of high resolution mass spectrometer. The column used is purchased from Waters Acquity UPLC HSS T3 column (1.8 μm 2.1 × 100 mm). The experimental methods were as follows. The sample (50 mg) was added to 1 ml extract buffer containing an internal standard (methanol/acetonitrile, 2:2, v/v; internal standard concentration 20 mg/L), swirled for 30 s. Then mixture was treated with ultrasound for 10 min in an ice water bath and standing for 1 h at −20°C. The homogenate was centrifuged at 4°C and 12,000 × *g* for 15 min. A sample (500 μl) of the supernatant was carefully removed to the tube and dried in a vacuum concentrator. An aliquot (160 μl) of the extract (acetonitrile/water, 1:1, v/v) was added to the dried metabolites for resolution. After vortexing for 30 s being treated with ultrasound for 10 min in an ice water bath, the solution was centrifuged at 4°C and 12,000 × *g* for 15 min. A sample (120 μl) of the supernatant was transferred to a 2 ml injection flask and mixed with 10 μl from each sample for the QC sample analysis. Kyoto Encyclopedia of Genes and Genomes (KEGG) database was used for metabolites annotation and enrichment analysis.

### Determination of total phenols, flavonoids, and ascorbic acid content

To measure the total phenols in peel and pulp, 100 mg of freeze-dried flesh samples was homogenized with 1.5 ml of 60% aqueous ethanol and vibrated for 2 h under sonication, then centrifuged at 25°C and 12,000 × *g* for 10 min. Total phenols content was determined using the Folin-Denis method described previously with some modifications (34). The sample extract (10 μl) was mixed briefly with 50 μl Folin-Ciocalteu phenol reagent, 10 μl distilled water and 50 μl reagent were prepared as the blank, and the mixture were kept in the dark for 3 min. Then 50 μl of 10% Na_2_CO_3_ and 90 μl distilled water was added, adjust the total volume to 200 μl. The sample mixture was incubated at room temperature for 60 min, and then its absorbance was measured at 760 nm by NanoDrop 2000C (Thermo Scientific, USA). Gallic acid was used as a standard and total phenols was expressed as mg GAE/g FW extract.

Total flavonoids content was determined according to the method described by Kim et al. (35). After extraction, 15 μl of 5% NaNO_2_ were added to a 50 μl extract in a volumetric flask, and the mixture was kept for 6 min under dark at room temperature. Then, 30 μl of 10% Al (NO_3_)_3_ was added to the mixture and incubated for 6 min again. At last, 105 μl of 1 M NaOH was added. After incubating for 15 min at room temperature, the absorbance was measured at 510 nm. Results were expressed as mg of rutin equivalents (RE) per gram of FW (mg RE/g FW) for the total flavonoids content.

Measurement of ascorbic acid content was performed according to Chiaiese et al. (36). Fresh sample of 0.5 g was homogenized in 5 ml of 5% (w/v) TCA (trichloroacetic acid). After centrifugation at 6,000 rpm for 15 min, 1 ml of the supernatant was incubated with the mixture of 1 ml of TCA, 0.5 ml of 0.4% phosphoric acid-ethanol (w/v), 1 ml of 0.5% bathophenanthroline-ethanol (w/v) and 0.5 ml of 0.03% FeCl_3_-ethanol (w/v) for 60 min at 30°C. The ascorbic acid content was calculated at 534 nm and expressed in mg⋅g^–1^ FW.

### Assays of antioxidant activity

Ferric reducing/antioxidant power, DPPH (1,1-diphenyl-2-picrylhydrazyl radical) and ABTS (2,2′-azinobis 3-ethylbenzothiazoline-6-sulfonic acid) assays were conducted by the method in a previous report (32). FRAP reagent was prepared by mixing 200 ml acetate buffer (300 mM, pH 3.60), 20 ml of FeCl_3_⋅6H_2_O solution (20 mM) and 20 ml TPTZ solution (10 in 40 mM HCl). Then 3.80 ml FRAP reagent was added to 200 μl of peel or pulp extract. After 30 min at room temperature, the absorbance of extracts and reference substances Trolox was detected at a wavelength of 590 nm by NanoDrop 2000C (Thermo Scientific, USA). For DPPH assay, 3.50 ml of DPPH was added into 500 μl ethanol extracts. After 30 min for darkness, the absorbance was detected at wavelength of 517 nm. For ABTS assay, fruit extracts (40 μl) were allowed to react with 3.90 ml of the ABTS radical solution under dark conditions for 10 min, and then the absorbance at 734 nm was measured. The results of ABTS, DPPH, and FRAP were represented as μmol of Trolox equivalent per gram fresh weight (μmol Trolox/g FW) for peels and pulps. Three repeats were performed for one sample.

### Statistical analysis

All tests were conducted in triplicate. Statistical analysis was performed using IBM SPSS Statistics 22.0. Significant differences of the contents of total phenols, total flavonoids, ascorbic acid, and the antioxidants activities of FRAPs, DPPH and ABTS in peel and pulp of the four different citrus varieties were calculated. Tukey’s test was performed by using one-way analysis of variance (ANOVA) at the 5% level (*P* < 0.05). The Pearson correlation analysis was also performed by SPSS 22.0 at *P* < 0.05 for determination of the correlations among the contents of total phenols, total flavonoids, and vitamin C and the antioxidant activities of citrus peel and pulp, respectively.

## Results

### Biological characteristics and phylogenetic analysis of two local citrus varieties

**FIGURE 1:**
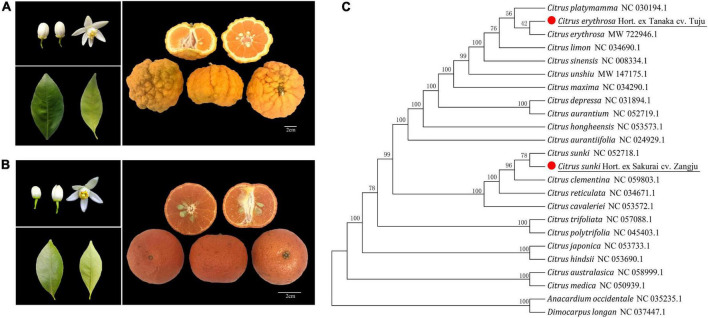
Biological characteristics and phylogenetic analysis of two local citrus varieties. **(A)** Biological characteristics of leaves, flowers, and fruits of Zangju. **(B)** Biological characteristics of leaves, flowers, and fruits of Tuju. **(C)** Phylogenetic analysis of Zangju and Tuju with other citrus species. The phylogenetic tree of 24 citrus and related species was conducted using chloroplast DNA sequences data.

Zangju (*Citrus sunki* Hort. ex Sakurai cv. Zangju), also known as Dengrongzhoupigan, is mainly distributed in Derong county of Ganzi Tibetan Autonomous Prefecture of Sichuan province, with a longitude of 99° 30′ 15″ and a latitude of 28° 61′ 27″. The planting area of Zangju in the county is about 1,000 μ, mainly distributed in Bendu and Guxue towns. The biological characteristics of Zangju are as follows (Figure 1A and Table 1): trees of Zangju are moderately large and vigorous, with semicircular crowns. Young shoots of Zangju are light green with simple leaves, wedge-shaped leaf base, linear wing leaves with an acuminate tip, entire leaf margins. Zangju flowers occur singly or in clusters, and are complete flowers with 5–6 white petals, medium pollen, filaments partially united, erect light-yellow style, and the length yellow anthers are longer than stigma. Zangju fruit is slightly flattened in shape with large fruit size for a mandarin, with deep radiating grooves and rough in the rind, dense oil cells. The single weight of fruit is 150 g, the transverse and longitudinal diameter is 7.89 and 5.70 cm, and the fruit shape index is 0.72; the rind is orange-yellow, with the thickness 6–8 mm, rather large open core, easy to peel, segments 9–10, numerous oval monoembryonic seeds (13–15). The flesh is deep orange in color with a moderately fine texture, fragrant and rich in juice. Fruit quality characteristics were measured for Zangju, including the soluble solids content (11.3°Brix) and total organic acid content (1.03%), solid-acid ratio of 11.07, slightly sour taste. The fruit of Zangju matures at late November, and can be picked until March.

**TABLE 1:** Fruit characteristics and quality of Zangju and Tuju.

Index	Segment number	Seed number	Single fruit weight (g)	Equatorial diameter (cm)	Fruit height (cm)	Fruit shape index	TSS (Brix)	Titratable acidity (%)	TSS/TA ratio
Zangju	9.83 ± 0.43	13.75 ± 0.96	147.01 ± 7.10	7.89 ± 0.42	5.70 ± 0.39	0.72 ± 0.05	11.30 ± 0.54	1.03 ± 0.09	11.07 ± 1.15
Tuju	10.67 ± 1.03	13.25 ± 1.26	47.04 ± 4.88	4.43 ± 0.2	3.76 ± 0.01	0.85 ± 0.05	14.48 ± 0.78	0.65 ± 0.06	22.64 ± 3.22

Data are expressed as mean ± SE.

Tuju (*Citrus erythrosa* Hort. ex Tanaka cv. Tuju), is mainly distributed in Jiukeng town, Chunan county of Zhejiang province, with a longitude of 118° 38′ 42″ and a latitude of 29° 38′ 15″. There is little amount of cultivation of Tuju in the local area, and it has been developed and utilized in recent years to process tangerine peel, small green tangerine, tea wine, etc. Hence, the cultivation of Tuju has been gradually expanded. The biological characteristics of Tuju are as follows (Figure 1B and Table 1): trees of Tuju are evergreen, moderate tree with vigor good cold resistance. Young shoots of Tuju are light green with simple leaves, showing similar leaf characteristics with Zangju. Tuju flowers occur singly or in clusters, and are complete flowers with 5 white petals, medium pollen, filaments partially united, erect green-yellow style, and the length yellow anthers are longer than stigma. Tuju fruit is globe in shape, no radiating grooves and smooth in the rind, dense, and fine oil cells. The single weight of fruit is 47 g, the transverse and longitudinal diameter is 4.43 and 3.76 cm, and the fruit shape index is 0.85; the rind is red and orange, with the thickness 2-3 mm, rather little open core, easy to peel, segments 10–11, numerous oval polyembryonic seeds (12–15). The flesh is red and orange in color with a moderately fine texture, fragrant and rich in juice. The soluble solids content of fruit is 14.5°Brix, total organic acid content is 0.65%, and solid-acid ratio of juice is 22.64. The fruit of Tuju matures at early December with sweet and moderate sour taste.

Chloroplast genomes of fruit trees play an important role in phylogenetic studies. To understand the evolutionary relationships of Zangju and Tuju with other species, the complete chloroplast genome sequences of 22 species of the genus *Citrus*, including typical mandarin, sweet orange, lemon, pomelo, sour orange, and citron, were used to construct a phylogenetic relationship tree, with *Anacardium occidentale* and *Dimocarpus longan* as the outgroups (Figure 1C). The phylogenetic analysis revealed that Zangju belonged to *C. reticulata* and was phylogenetically closest with sour mandarin *C. sunki*, whereas Tuju was a kind of red orange that closely clustered with *C. erythrosa*. The phylogenetic analysis also indicated that Zangju and Tuju from the south and north of China have a distant phylogenetic relationship.

### Phytochemical metabolites analysis in the peel and pulp of ZG and TG

#### Metabolites profiles in the peel and pulp

**FIGURE 2:**
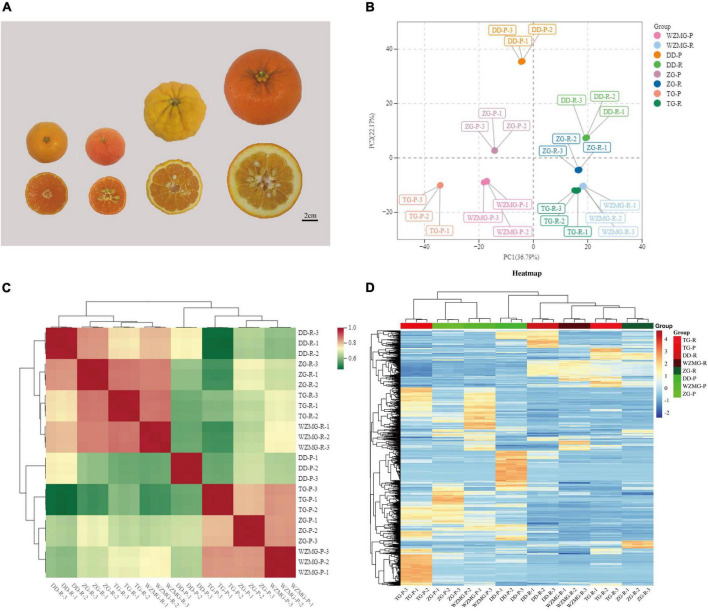
Samples and metabolites profiles in the peel and pulp. **(A)** Samples of Zangju (ZG), Tuju (TG), Daidai (DD), and satsuma mandarin (WZMG). **(B)** Principal component analysis (PCA) score plot of the first and second principal components of all the samples. **(C)** Heatmap of biological replicate sample correlation analysis. **(D)** Heatmap of the total 1,126 detected metabolites of all groups.

To better understand the nutritional and medicinal differences among Zangju (ZG), Tuju (TG), Daidai (DD), and satsuma mandarin (WZMG), we performed widely targeted UPLC-MS/MS-based non-targeted metabolite profiling of these four species. The satsuma mandarin WZMG, the most widely cultivated variety in China (37) and DD fruit (*Citrus aurantium* L. var. *daidai*) contained the higher amounts of flavonoids and medicinal components than other citrus species according (38). Hence, here we used the sour orange DD and satsuma mandarin WZMG as controls (Figure 2A).

To obtain a clear overview of the clear separation among the fruit peel and pulp of the four citrus varieties, we conducted an unsupervised principal component analysis (PCA) (Figure 2B), revealing that the first and second principal components (PC1 and PC2) displayed 36.79 and 22.17% of the variation, respectively. PCA analysis of metabolomics data showed that 24 samples of fruit peel and pulp were significantly separated into 8 groups (Figure 2B), indicating that the fruit samples from every group exhibited different metabolic characters and the metabolomics data were reliable. We performed an intragroup correlation analysis and found that biological replicates of the samples from the same variety were highly correlated (*r*^2^ > 0.7), whereas peel and pulp samples showed significant differences (Figure 2C). As shown in Figure 2D and Supplementary Table 1, 1,126 metabolites were totally identified, of which there were a large number of secondary metabolites likely to contribute to the medicinal value, and some primary metabolites are related to the nutritional quality. Similar to the previous studies (24, 25), it was also shown that the relative abundance of most of the detected metabolites in the peel of TG and ZG fruits were significantly higher than that in the pulp (Figure 2D).

**FIGURE 3:**
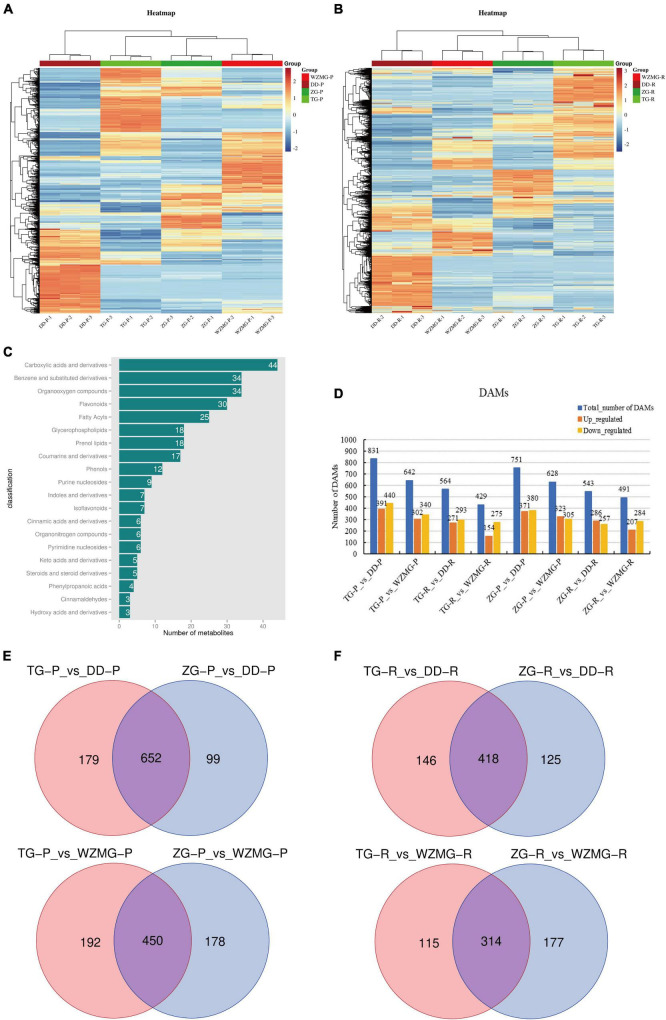
Comparative analysis of differentially accumulated metabolites (DAMs) in the pulp and peel of ZG, TG, DD, and WZMG. **(A)** Heatmap of the different metabolites among the peel (P) of ZG, TG, DD, and WZMG. **(B)** Heatmap of the different metabolites among the pulps (R) of ZG, TG, DD, and WZMG. **(C)** Top 20 classifications of all different metabolites. **(D)** The number of up- or down-regulated DAMs in different comparison groups. **(E)** The DAMs number of TG-P (ZG-P) vs. DD-P and TG-P (ZG-P) vs. WZMG-P shown in Venn diagrams. **(F)** The DAMs number of TG-R (ZG-R) vs. DD-R and TG-R (ZG-R) vs. WZMG-R shown in Venn diagrams.

A total of 1,042 differentially accumulated metabolites (DAMs) were detected in the peel (Figure 3A), and 985 DAMs were detected in the pulp (Figure 3B). TG peel contains most secondary metabolites with higher abundance, followed by DD, WZMG, and ZG (Figure 3A and Supplementary Table 1). Similarly, TG pulp contains most abundant secondary metabolites, followed by DD, ZG, and WZMG (Figure 3B and Supplementary Table 1). Additionally, among all the DAMs, carboxylic acids and derivatives, benzene and substituted derivatives, organooxygen compounds, flavonoids, fatty acyls glycerophospholipids, prenol lipids, coumarins and derivatives, phenols, etc. were listed in the top 20 classification (Figure 3C). As shown in Figure 3D, based on FPKM (fragments per kilobase million) data, there were 831, 642, 564, and 429 DAMs were identified in the groups of TG_P vs. DD_P, TG_P vs. WZMG_P, TG_R vs. DD_R, TG_R vs. WZMG_R, respectively. Furthermore, 751, 628, 543, and 491 DAMs were identified in the groups of ZG_P vs. DD_P, ZG_P vs. WZMG_P, ZG_R vs. DD_R, and ZG_R vs. WZMG_R, respectively (Figure 3D). Compared with DD peel, TG peel contained more distinct DAMs than ZG peel, 652 DAMs in common, and 450 conserved DAMs were found between TG and ZG peels vs. WZMG peel (Figure 3E). Compared with DD pulp, there were 418 DAMs in common between TG and ZG pulp, whereas, compared with WZMG pulp, ZG pulp contained more distinct DAMs than TG pulp, with 314 conserved DAMs (Figure 3F). These results also revealed significant diversity and specificity of the metabolites in the peel and pulp of TG and ZG fruits.

#### Analysis of the nutrition and antioxidant related DAMs in TG and ZG

**FIGURE 4:**
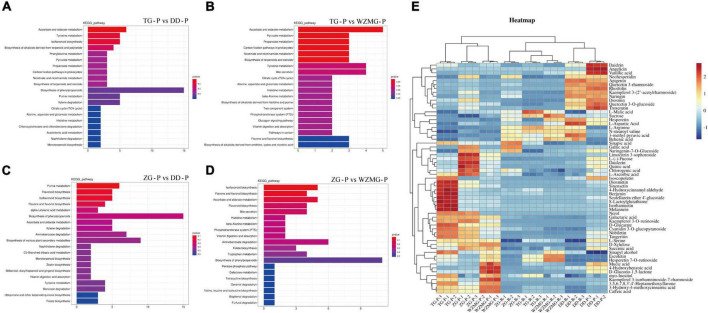
Analysis of the DAMs and nutrition and antioxidant metabolites in ZG and TG. KEGG pathway enrichment analysis of DAMs in groups TG-P vs. DD-P **(A)**, TG-P vs. WZMG-P **(B)**, ZG-P vs. DD-P **(C)**, and ZG-P vs. WZMG-P **(D)**. **(E)** Heatmap of the key DAMs in the peel and pulp.

In order to explore the metabolite function of TG and ZG fruits, we further analyzed the KEGG enrichment pathway of the DAMs. The results showed that, compared with the peel and pulp of DD and WZMG, the DAMs in TG and ZG fruit were mainly enriched in ascorbate and aldarate metabolism, tyrosine metabolism, flavonoid biosynthesis, isoflavonoid biosynthesis, biosynthesis of alkaloids derived from terpenoid and polyketide, flavone and flavonol biosynthesis, biosynthesis of phenylpropanoids, phenylalanine metabolism, pyruvate metabolism, tryptophan metabolism, starch and sucrose metabolism, biosynthesis of terpenoids and steroids, xylene degradation, and citrate cycle (TCA cycle) pathway (Figures 4A–D and Supplementary Figure 1). According to the KEGG enrichment pathway analysis of the DAMs in the peel (Figures 4A–D) and pulp (Supplementary Figure 1), relative abundance of main DAMs in the key pathways was displayed in the heat map. The major DAMs in the pulp and peel included some primary and secondary metabolites related to nutrition, such as sugars, amino acids, organic acids, and antioxidant activity, such as flavonoids, flavanones, isoflavonoids, vitamin C, and phenolic acids (Figure 4E). As shown in the heat map, both TG and ZG peel contains several DAMs with significantly higher abundance, whereas, less DAMs with high abundance were found in the pulp of TG and ZG.

The antioxidant activity and medicinal value of citrus fruits are mainly related to flavonoids, phenolic acids, and vitamin C (17, 21). Based on the metabolome data, we analyzed the relative abundance of ascorbic acid and major flavonoids and phenolic acids in the peel and pulp of ZG and TG. The relative abundances of ascorbic acid and key flavonoids and phenolic acids are listed in Table 2. The relative abundance of ascorbic acid in ZG and TG was higher than that in DD and WZMG, except for the content in the pulp of TG (Table 2). Compared with DD and WZMG peel, the relative abundance of some key flavonoids and phenolic acid, such as diosmetin, sinensetin, tangeritin, nobiletin, quercetin (quercetin 5,7,3′,4′-tetramethyl ether), scutellarein (scutellarein 6,7-dimethyl ether 4′-glucoside), sinapic acid, etc. were significantly higher in TG peel (Table 2). Furthermore, ZG peel showed relatively higher abundance of flavonoids and phenolic acid, such as diosmetin, sinensetin, tangeritin, nobiletin and caffeic acid, gallic acid, sinapic acid, etc. compared with DD and WZMG peel (Table 2). Additionally, both the relative abundance of naringin and neohesperidin in ZG peel and pulp are higher than that in WZMG. Moreover, TG pulp recorded higher relative abundance of sinensetin, tangeritin, heptamethoxyflavone (3,5,6,7,8,3′,4′-heptamethoxyflavone), nobiletin, caffeic acid, sinapic acid, chlorogenic acid, etc. than DD and WZMG pulp (Table 2). ZG pulp showed relatively higher abundance of key flavonoid and phenolic acid, such as tangeritin, caffeic acid, gallic acid, sinapic acid, chlorogenic acid, etc. compared to DD and WZMG pulp. Additionally, ZG and TG peel and pulp recorded relatively higher content of hesperidin than DD, but lower than the previously reported the highest hesperidin content in WZMG (39). In conclusion, these findings suggest that the peel and pulp of these two local varieties ZG and TG are rich of some key flavonoids and phenolic acid.

**TABLE 2:** Represents the log_2_FC of the main flavonoids, phenolic acid, and vitamin C in fruit peel and pulp between different groups.

Metabolites name	Category	Log2 of fold change of different groups of fruit peel				Log2 of fold change of different groups of fruit pulp			
		TG-P vs. DD-P	TG-P vs. WZMG-P	ZG-P vs. DD-P	ZG-P vs. WZMG-P	TG-R vs. DD-R	TG-R vs. WZMG-R	ZG-R vs. DD-R	ZG-R vs. WZMG-R
Ascorbic acid	Vitamin C	1.61	1.29	0.93	1.25	-0.17	-0.31	1.11	0.97
Apigenin	Flavonoids	-5.20	-0.03	-2.63	2.54	-7.50	0.78	-4.00	4.28
Diosmetin	Flavonoids	2.37	3.34	0.85	1.82	-1.80	0.81	-2.39	0.23
Diosmin	Flavonoids	-1.65	1.17	-0.47	2.35	-4.39	-0.30	-3.05	1.05
Naringin	Flavonoids	-4.97	-1.40	-1.85	1.72	-6.40	-1.14	-1.33	3.92
Hesperetin	Flavonoids	-2.30	0.50	-2.03	0.77	-3.66	-3.39	-0.84	-0.57
Hesperidin	Flavonoids	3.48	-0.48	2.75	-1.21	4.13	-0.74	1.91	-2.96
Neohesperidin	Flavonoids	-3.53	-0.61	-0.03	2.89	-4.52	-1.22	-0.08	3.21
Sinensetin	Flavonoids	3.15	2.15	1.79	0.79	2.16	3.00	-0.62	0.22
Tangeritin	Flavonoids	1.51	1.84	1.19	1.51	2.88	3.57	1.39	2.08
Heptamethoxyflavone	Flavonoids	2.74	0.04	1.34	-1.37	2.50	0.23	-0.10	-2.37
Kaempferol	Flavonoids	-2.91	1.10	-0.92	3.09	-5.64	3.84	-1.61	7.86
Nobiletin	Flavonoids	1.11	1.30	0.75	0.94	1.68	3.13	-0.10	1.35
Quercetin	Flavonoids	4.32	2.67	1.54	-0.98	7.12	3.52	0.00	-6.79
Scutellarein	Flavonoids	2.25	3.37	-0.80	0.31	-2.98	-1.43	-0.68	0.87
Troxerutin	Flavonoids	-4.33	-0.45	-1.43	2.45	-7.59	0.92	-2.58	5.93
Caffeic acid	Phenolic acid	1.67	-0.42	1.36	-0.73	1.29	0.10	0.65	-0.54
Gallic acid	Phenolic acid	-4.87	-1.48	2.48	5.87	-2.35	-0.64	3.96	5.67
Vanillic acid	Phenolic acid	-6.50	-2.92	-3.81	-0.24	-6.11	-4.96	-2.38	-1.23
Sinapic acid	Phenolic acid	2.01	0.79	1.40	0.18	0.76	1.31	1.85	2.39
Chlorogenic acid	Phenolic acid	0.04	-0.03	0.01	0.26	0.73	0.66	0.62	0.87
4-Hydroxybenzoic acid	Phenolic acid	-2.49	-3.47	-1.79	-2.76	-1.59	-0.63	-2.37	-1.41

Importantly, besides the polyphenols listed in Table 2, metabolite profiling showed that some secondary metabolites, such as bergenino was only detected in ZG and TG, with the highest relative content in the peel of TG (Supplementary Table 1). Furthermore, 5-hydroxyferulate, harpagoside, 1-naphthol, and methylenedioxycinnamic acid were only found in the peel of ZG (Supplementary Table 1). Therefore, further research on the extraction and application of these special metabolites in ZG and TG is needed in the future.

#### Comparative analysis of the contents of total phenols, total flavonoids, and vitamin C in TG and ZG fruit

**FIGURE 5:**
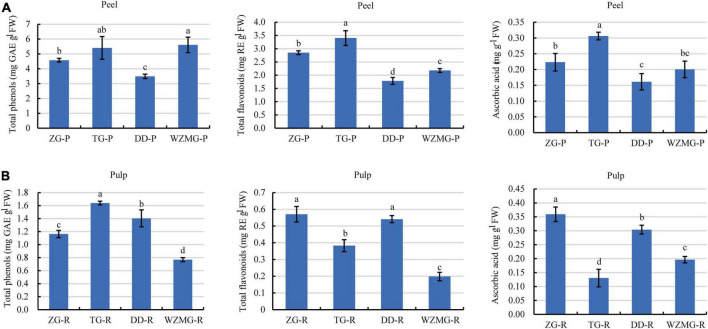
Comparative analysis of the contents of total phenols, total flavonoids, and ascorbic acid in peel and pulp. **(A)** Contents of total phenols, total flavonoids, and ascorbic acid in ZG-P, TG-P, DD-P, and WZMG-P. **(B)** Contents of total phenols, total flavonoids, and ascorbic acid in ZG-R, TG-R, DD-R, and WZMG-R. The different letters in each bar indicate significant differences by Tukey’s test (*P* < 0.05). Total phenols and total flavonoids were expressed as gallic acid equivalents (GAE) and rutin equivalents (RE), respectively. FW, fresh weight.

Citrus peel is a rich source of naturally occurring antioxidants (39). Antioxidant activity of citrus peel is due to the abundance of phenols, flavonoids, and ascorbic acid (25, 39). Here, as shown in Figure 5A, satsuma mandarin WZMG peel has the highest total phenols content of 5.61 mg GAE/g FW, followed by TG peel (5.40 mg GAE/g FW), ZG peel (4.58 mg GAE/g FW), DD peel (3.50 mg GAE/g FW). Furthermore, TG peel has highest content of total flavonoids of 3.40 mg RE/g FW, followed by ZG (2.85 mg RE/g FW), WZMG (2.18 mg RE/g FW), and DD (1.78 mg RE/g FW). The TG peel recorded the highest total ascorbic acid content (0.31 mg/g FW), followed by ZG (0.22 mg/g FW), WZMG (0.20 mg/g FW), and DD (0.16 mg/g FW).

Moreover, the TG pulp contained the highest content of total phenols of 1.64 mg GAE/g FW (Figure 5B), followed by DD pulp (1.41 mg GAE/g FW), ZG pulp (1.16 mg GAE/g FW), WZMG pulp (0.77 mg GAE/g FW). ZG pulp recorded the highest total flavonoids content (0.57 mg RE/g FW), followed by DD pulp (0.54 mg RE/g FW), TG pulp (0.38 mg RE/g FW) and WZMG pulp (0.20 mg RE/g FW). The contents of total flavonoids and phenols in the peel of the four different citrus varieties were significantly higher than that in the pulp, which is consistent with the previous studies (39). Additionally, ZG pulp has the highest ascorbic acid content (0.36 mg/g FW), followed by DD pulp, WZMG pulp, and TG pulp (Figure 5B).

#### Comparative analysis of antioxidant activities of the peel and pulp of TG and ZG fruits

**FIGURE 6:**
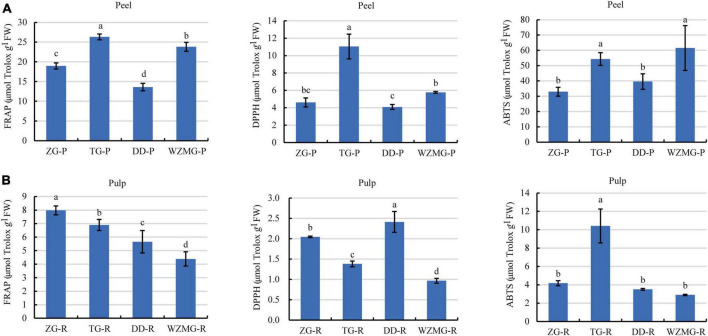
Comparative analysis of antioxidant activity of peel and pulp. **(A)** FRAP activity, DPPH scavenging activity, and ABTS scavenging activity of ZG-P, TG-P, DD-P, and WZMG-P. **(B)** FRAP activity, DPPH scavenging activity, and ABTS scavenging activity of ZG-R, TG-R, DD-R, and WZMG-R. The different letters in each bar indicate significant differences by Tukey’s test (*P* < 0.05). FRAP, DPPH, and ABTS value were expressed as μmol Trolox equivalents/g FW. FW, fresh weight.

Phenolic compounds, including flavonoids and phenolic acids, are known to be responsible for antioxidant activity of different fruits, and fruits with higher total phenolic content generally showed stronger antioxidant capacity (40). The antioxidants activities of peel and pulp of ZG and TG were evaluated through FRAPs, DPPH, and ABTS assays. The FRAP activity of the four citrus varieties peels ranged from 13.60 to 26.31 μmol Trolox/g FW. The TG peel displayed the strongest activity, followed by satsuma mandarin WZMG peel, DD peel, and ZG peel (Figure 6A). The DPPH value of the four citrus varieties peels ranged from 4.62 to 11.04 μmol Trolox/g FW, where TG peel showed the strongest DPPH scavenging activity, followed by WZMG peel, ZG peel, and DD peel (Figure 6A). Furthermore, ABTS scavenging activity of the four citrus varieties peels ranged from 32.95 to 61.51 μmol Trolox/g FW, where WZMG peel exhibited the strongest activity, and the rest were in descending order: TG peel > DD peel > ZG peel (Figure 6A). In conclusion, these results suggest that TG peel exhibits the stronger antioxidant activity.

Further, FRAP activity of the four citrus varieties pulp ranged from 4.39 to 7.98 μmol Trolox/g FW, where ZG Pulp showed the strongest FRAP activity, followed in the order TG pulp > DD pulp > WZMG pulp (Figure 6B). The DPPH scavenging activities of the four citrus pulps ranged from 0.97 to 2.41 μmol Trolox/g FW, where DD pulp displayed strongest activity, followed by ZG pulp, TG pulp, and WZMG pulp (Figure 6B). The value of ABTS scavenging activity were between 3.50 and 10.41 μmol Trolox/g FW, where TG pulp showed the significantly higher activity, followed by ZG pulp, DD pulp, and WZMG pulp (Figure 6B). These results indicate that TG and ZG pulps exhibit relatively stronger antioxidant activities.

#### Correlation analysis between antioxidant activities and contents of total phenols, flavonoids, and vitamin C in peel and pulp

Recently, few studies concluded that total antioxidant activity of citrus fruits are mainly attributed to phenolic compounds and vitamin C, though there is disagreement as to which compound is the major contributor (28). To identify the chemical compounds which contribute to the antioxidant activity of the four citrus fruits, Pearson’s correlation coefficients between the contents of total phenols, total flavonoids, and vitamin C and the antioxidant activity of citrus peel and pulp were analyzed.

As shown in Table 3, the antioxidant activity (FRAP) of the peels of the four citrus varieties had the strongest correlation with the total phenols content (*r* = 0.873, *P* < 0.01), followed by the vitamin C content (*r* = 0.782, *P* < 0.01) and total flavonoid content (*r* = 0.673, *P* < 0.05). The correlation between DPPH value and vitamin C content was the strongest (*r* = 0.829, *P* < 0.01), followed by the correlation with total flavonoids (*r* = 0.740, *P* < 0.01), but there was no significant correlation with total phenols content. However, ABTS value in peel showed no significant correlation with the content of total flavonoids, total phenols, and vitamin C. For pulp, FRAP of the four citrus varieties had the strongest correlation with the content of total flavonoids (*r* = 0.726, *P* < 0.01), but displayed no significant correlation with the content of total phenols and vitamin C. DPPH value of pulp also had the strongest correlation with the content of total flavonoids (*r* = 0.857, *P* < 0.01), followed by the content of vitamin C (*r* = 0.735, *P* < 0.01), but had no significant correlation with the content of total phenols. However, ABTS value of pulp showed the strongest significant correlation with total phenols content (*r* = 0.731, *P* < 0.01). These results suggest that the main antioxidant components in citrus fruits are phenols, flavonoids, and vitamin C, which is consistent with previous reports (8).

**TABLE 3:** Correlation analysis of total phenols, total flavonoids, and vitamin C content and antioxidant activity in peel and pulp.

Citrus tissues	Compounds	FRAP	DPPH	ABTS
Peel	Total flavonoids	0.673*	0.740**	0.088
	Total phenols	0.873**	0.485	0.472
	Vitamin C	0.782**	0.850**	0.172
Pulp	Total flavonoids	0.726**	0.857**	0.155
	Total phenols	0.467	0.437	0.731**
	Vitamin C	0.341	0.735**	-0.602*

*Correlation is significant at the 0.05 level. **Correlation is significant at the 0.01 level (two-tailed).

## Discussion

In the present study, the morphological characteristics, phylogenetic relationships, phytochemical profiling, and antioxidant activities of two local citrus varieties, Zangju (ZG) and Tuju (TG), were systematically analyzed for the first time. We found that ZG fruit grown in Sichuan province has a little acidic fruit flavor and phylogenetically closest relationship with *C. sunki*, whereas TG grown in Zhejiang province has sweet fruit taste and phylogenetically closest with *C. erythrosa*. Fruits qualities and flavors of ZG and TG are consistent with the dietary habits of local residents, which is also one of the reasons why these two citrus varieties have been cultivated so far. Both Sichuan and Zhejiang provinces are the main producing areas of citrus in China and rich in citrus varieties (41). These two local varieties are still be preserved and cultivated in citrus producing areas for a long time, indicating that their fruits may contain some metabolites with beneficial effects on human health besides the characteristic flavors.

Citrus fruits are highly nutritious, containing many primary metabolites, which are important sources of essential nutrients, and secondary metabolites, which form an excellent source of bioactive substances and exhibit potent health-promoting effects (42). Chemical profiles, such as primary and secondary metabolites related to taste, color, and health benefits, are significantly different depending on the citrus varieties, leading to different general quality parameters and antioxidant activities (43–45). Several previous reports showed that the highest levels of total phenols and total flavonoids were found in mandarin (25–29). The phytochemical metabolites analysis showed that TG peel and pulp recorded higher abundance of some important secondary metabolites compared with the controls DD and mandarin WZMG. The relative abundance of flavonoids, such as tangeritin, sinensetin, diosmetin, and nobiletin, in the peel and pulp of ZG and TG were higher than that in sour range DD and mandarin WZMG. Nobiletin and tangeritin are the primary and most widely distributed flavonoids for their bioactivities in the peels of different citrus species (46, 47). Mandarin peel is rich in nobiletin as compared to other citrus species, and the concentration of isolated nobiletin varied significantly among the peel extracts of mandarin, sweet orange, white grapefruit and lime with striking different values of 202.91, 73.15, 18.13, and 0.09 μg/ml, respectively (48). However, our results showed that the contents of nobiletin and tangeritin in both the peel and pulp of ZG and TG are relative higher than that in mandarin WZMG, especially in the pulp. Furthermore, both the relative content of naringin and neohesperidin in ZG peel and pulp are significantly higher than that in WZMG, similar with that in two wild zhoupigan varieties (8). These relatively abundant flavonoids in ZG and TG, including naringin, neohesperidin, tangeritin, sinensetin, diosmetin, and nobiletin, are the main flavonoids in most citrus, which is consistent with previous reports (27, 32). Hence, higher contents of these flavonoids with important medicinal value (49) will furtherly increase the economic value of ZG and TG fruits. Phenolic acid, such as caffeic acid, chlorogenic acid, and sinapic acid, contents are abundant in ZG and TG, except for gallic acid, which is contrary to some previously published reports (50–52). For example, ferulic acid was identified as the most abundant and caffeic acid as the least abundant phenolic acid in kinnow peel extract (53). Ferulic acid was quantified as a major phenolic acid and caffeic acid as minor in peels of citrus fruits including lemons, oranges and grapefruits, the levels of which were significantly higher than those of peeled fruits (50). The level of chlorogenic, caffeic, and ferulic acid were the highest of phenolic acids in citrus hybrids peels from China (51). Similarly, chlorogenic acid is the phenolic acid with highest abundance in ZG, TG, DD, and WZMG (Supplementary Table 1). However, gallic acid is identified as the major phenolic acid in all grapefruit tested (52). Besides the major flavonoids and phenolic acid listed above, some secondary metabolites, such as bergenino was only detected in ZG and TG, with the highest relative content in the peel of TG. Furthermore, 5-hydroxyferulate, harpagoside, 1-naphthol, and methylenedioxycinnamic acid were only found in the peel of TG. Hence, it is necessary to further study the extraction and application of these special metabolites in ZG and TG in the future.

In general, citrus fruits are considered as one of the natural resources of antioxidants, which contain an appreciable amount of ascorbic acid, flavonoids, and phenols compounds (54–56). These active antioxidants have a wide variety of biological activities of importance to human health (3, 11–15). However, due to the dissimilarities in composition of antioxidants, antioxidant activities of fruit varies among different citrus species and tissues (39, 50). All the four citrus peels presented higher total flavonoids and phenols content than pulps, which is consistent with the previous reports (57, 58). Though antioxidants, such as flavonoids, phenols, in DD and WZMG are higher than some citrus varieties (27), our results showed that the peel or pulp of ZG and TG have higher contents of total flavonoids, total phenols, and vitamin C than DD and WZMG, indicating that both ZG and TG have much more richer antioxidants that provide human health benefits, such as antioxidative, anti-inflammatory, anticancer, and cardiovascular protective activities (59). Additionally, it is previously reported that DD and WZMG peels or pulps have stronger antioxidant activity than some other citrus varieties (25, 60), and it has also been reported that DD has the strongest antioxidant activity in several sour oranges (61). However, compared to DD and WZMG, TG peel and ZG pulp have the strongest FRAP activity and a higher DPPH scavenging activity, and TG pulp has the highest ABTS scavenging activity. This may be due to the relatively higher content of some major flavonoids in TG and ZG, such as nobiletin and tangeritin. Previously, Chen et al. reported that the orange peel collected from China contained the highest content of nobiletin and tangeritin compared to orange peel collected from USA and showed highest ABTS activity and DPPH free radical scavenging activity (62). All the four citrus peels showed much more higher antioxidant activities than pulps, due to the significantly higher contents of total flavonoids, total phenols and ascorbic acid in the peels, and these results are similar with the results observed by Xi et al. (58) and Nogata et al. (63). In summary, based on the higher amount of antioxidant compounds and stronger antioxidant activities in the fruits, we inferred that these two local varieties could offer some medicinal value for human health.

In the present study, we found that both FRAP value of the peels and pulp of the four citrus varieties displayed the strongest correlation with the total phenols content and total flavonoids, respectively, and this observation is in accordance with the previous reports that a positive correlation between the polyphenols contents and the antioxidant activities of different citrus germplasms (64). However, our results showed that the DPPH value of peel and pulp recorded strong correlation with total flavonoids and vitamin C content, but no significant correlation with total phenols content. The antioxidant activity may not always strongly correlate with phenols compounds. This is due to the correlation between DPPH value and antioxidants is depending on the types of fruit (65). Furthermore, a total of 39 flavonoids were identified and quantified from 35 varieties of five types of citrus fruit by Wang et al. (66), and they revealed that the correlation between DPPH value and total phenolics is also depending on the tissues of citrus fruit. In another study, Toh et al. reported that ABTS activity of two varieties of pomelo fruit positively correlated with total phenolic content and total flavonoid content, except for ascorbic acid (52). Interestingly, we found that ABTS value in peel exhibited no significant correlation with the content of total flavonoids, total phenols, and vitamin C, and only displayed the significant correlation with total phenols content in pulp. However, Arena et al. (67) stated that phenolic compounds in citrus fruits contributed less than vitamin C in establishing the antioxidant power. Whereas, others reported that the antioxidant power is mainly governed mainly by phenolic compounds than ascorbic acid (68, 69). These distinctive differences in results may be due to cultivar types, maturity of fruit or the analytical methods used for estimation of antioxidant activity (70).

Our results show that the two local citrus varieties, ZG and TG, are rich in antioxidant related secondary metabolites and have strong antioxidant activities. Based on these results, we proposed that fruits of ZG and TG have certain medicinal value. Moreover, our findings will provide an important theoretical basis for the conservation and utilization of these two local citrus germplasm resources. Further research is needed to develop and utilize the medicinal value of these two local varieties to improve the economic benefits of citrus growers.

## Conclusion

In the present study, the biological, phylogenetic characteristics, and phytochemical profiles, antioxidants contents and antioxidant activities of two local citrus varieties, ZG and TG, were systematically demonstrated for the first time. The results showed that Zangju fruit had the characteristics of wrinkled skin and acidity, and grouped with *C. reticulata*, which had the closest phylogenetic relationship with sour orange *C. sunki*. Tuju is a kind of red orange with vermilion peel, small fruit and high sugar content, which is closely clustered with *C. erythrosa*. Phytochemical metabolites analysis showed that the relative content of some flavonoids and phenolic acid, such as tangeritin, sinensetin, diosmetin, nobiletin, and sinapic acid in the peel and pulp of both Zangju and Tuju were higher than that in sour range Daidai and satsuma mandarin. The contents of total flavonoids, total phenols, antioxidant activity (FRAP), DPPH and ABTS free radical scavenging capacity of peels of Zangju and Tuju were significantly higher than that in pulp. Compared with Daidai and satsuma mandarin, Zangju pulp and Tuju peel showed the strongest FRAP activity, and Tuju peel and pulp had the highest DPPH and ABTS value, respectively. Moreover, both the antioxidant activity in peel and pulp were significantly correlated with the contents of total phenols, total flavonoids, and ascorbic acid. The results suggested that Zangju and Tuju are rich in key antioxidants and have stronger antioxidant activity, indicating that they may have certain medicinal value and potential beneficial effects on human health.

## Data availability statement

The original contributions presented in this study are included in the article/Supplementary material, further inquiries can be directed to the corresponding author.

## Author contributions

LS, JX, and FK conceived and designed the experiments. JX, ZN, and JS collected the fruits. LS, JX, ZN, JS, and XH analyzed the fruit characteristics and quality. LS, JX, LW, and ZN performed the experiments of antioxidant activities. LS, LW, and N analyzed the data and elaborated the figures. LS wrote the manuscript. LS and N revised the manuscript. FK supervised the project. All authors read and approved the final manuscript.
