# Supplementary material for: Comprehensive studies of biological characteristics, phytochemical profiling, and antioxidant activities of two local citrus varieties in China
Source: Front Nutr. 2023 Jan 25;10:1103041. doi: 10.3389/fnut.2023.1103041 (PMC9905102; doi:10.3389/fnut.2023.1103041)
Supplement: Supplementary file 3 [file Table_1.DOCX]

Supplementary Material

# Supplementary Data

Table S1 All detected metabolites from the peel and pulp of ZG, TG, DD and WZMG fruits.

# Supplementary Figures


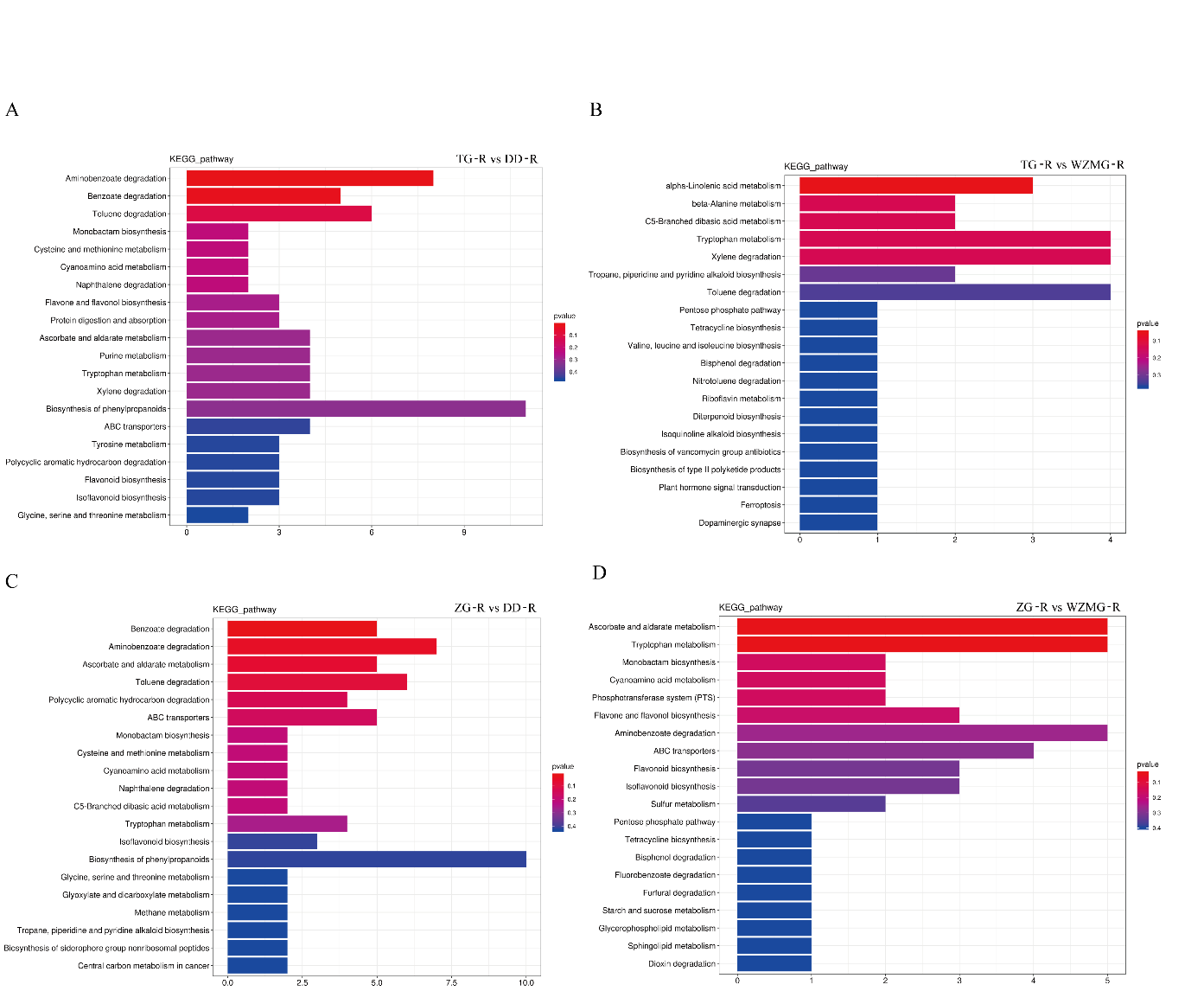


**Supplementary Figure 1.** Analysis of the KEGG enrichment of the DAMs in pulp of ZG and TG. KEGG enrichment pathways of DAMs in groups TG-R vs DD-R (A), TG-R vs WZMG-R (B), ZG-R vs DD-R (C) and ZG-R vs WZMG-R (D).
